# Oil blotting paper for formalin fixation increases endoscopic ultrasound‐guided tissue acquisition‐collected sample volumes on glass slides

**DOI:** 10.1002/cam4.7189

**Published:** 2024-05-06

**Authors:** Takuo Yamai, Kenji Ikezawa, Yusuke Seiki, Ko Watsuji, Yasuharu Kawamoto, Takeru Hirao, Kazuma Daiku, Shingo Maeda, Makiko Urabe, Yugo Kai, Ryoji Takada, Kaori Mukai, Tasuku Nakabori, Hiroyuki Uehara, Sayoko Tsuzaki, Ayumi Ryu, Satoshi Tanada, Shigenori Nagata, Kazuyoshi Ohkawa

**Affiliations:** ^1^ Department of Hepatobiliary and Pancreatic Oncology Osaka International Cancer Institute Osaka Japan; ^2^ Department of Gastroenterology Osaka General Medical Center Osaka Japan; ^3^ Department of Clinical Laboratory Osaka International Cancer Institute Osaka Japan; ^4^ Department of Diagnostic Pathology and Cytology Osaka International Cancer Institute Osaka Japan

**Keywords:** comprehensive genomic profiling, fine‐needle aspiration, fine‐needle biopsy, formalin fixation, oil blotting paper

## Abstract

**Objectives:**

Endoscopic ultrasound‐guided tissue acquisition (EUS‐TA) is used for pathological diagnosis and obtaining samples for molecular testing, facilitating the initiation of targeted therapies in patients with pancreatic cancer. However, samples obtained via EUS‐TA are often insufficient, requiring more efforts to improve sampling adequacy for molecular testing. Therefore, this study investigated the use of oil blotting paper for formalin fixation of samples obtained via EUS‐TA.

**Methods:**

This prospective study enrolled 42 patients who underwent EUS‐TA for pancreatic cancer between September 2020 and February 2022 at the Osaka International Cancer Institute. After a portion of each sample obtained via EUS‐TA was separated for routine histological evaluation, the residual samples were divided into filter paper and oil blotting paper groups for analysis. Accordingly, filter paper and oil blotting paper were used for the formalin fixation process. The total tissue, nuclear, and cytoplasm areas of each sample were quantitatively evaluated using virtual slides, and the specimen volume and histological diagnosis of each sample were evaluated by an expert pathologist.

**Results:**

All cases were cytologically diagnosed as adenocarcinoma. The area ratios of the total tissue, nuclear, and cytoplasmic portions were significantly larger in the oil blotting paper group than in the filter paper group. The frequency of cases with large amount of tumor cells was significantly higher in the oil blotting paper group (33.3%) than in the filter paper group (11.9%) (*p* = 0.035).

**Conclusions:**

Oil blotting paper can increase the sample volume obtained via EUS‐TA on glass slides and improve sampling adequacy for molecular testing.

## 1 INTRODUCTION

Pancreatic cancer is a major refractory cancer, with a 5‐year relative survival rate of 12%.^1^ The early diagnosis of pancreatic cancer presents major challenges,^2^, ^3^ and more than 80% of patients are diagnosed at an advanced stage and undergo conventional chemotherapy to increase their life expectancy.^4^ However, in 5–10% of patients, suspected pancreatic cancer based on imaging remains unconfirmed as malignant upon resection.^5^ Therefore, pathology‐based definitive diagnosis is recommended before chemotherapy and/or radiotherapy induction, regardless of the cancer stage.^6^, ^7^

Endoscopic ultrasound‐guided tissue acquisition (EUS‐TA) via fine‐needle aspiration (FNA) or fine‐needle biopsy (FNB) is the primary sampling method for diagnosing solid pancreatic lesions.^8^, ^9^, ^10^, ^11^ For patients with pancreatic cancer, the role of EUS‐TA has expanded from pathological diagnosis to obtaining adequate samples for molecular testing before initiating targeted therapies.^12^, ^13^, ^14^ The current National Comprehensive Cancer Network guidelines recommend the consideration of germline testing, gene profiling, and mismatch repair/microsatellite instability testing for metastatic pancreatic ductal cancer.^12^, ^15^ Moreover, pembrolizumab has been recently recommended for tumors exhibiting high microsatellite instability or a high tumor mutation burden; however, its efficacy is limited to a small number of cases.^16^, ^17^, ^18^, ^19^ In clinical practice, (CGP) tests have been reported to be beneficial for patients with pancreatic cancer.^20^, ^21^, ^22^, ^23^, ^24^ Notably, in Japan, CGP tests have been covered by Japanese health insurance since June 2019, highlighting the need for obtaining sufficient samples via EUS‐TA.

EUS‐FNB is superior to EUS‐FNA in providing adequate samples for CGP testing.^15^, ^25^, ^26^, ^27^ Although the technical excellence of 19G‐FNB has been demonstrated in clinical practice, its sampling adequacy remains limited. Hence, further efforts are required to improve the sampling adequacy for CGP tests.^28^, ^29^, ^30^, ^31^ Filter paper has been widely used as a receptacle for small biopsy specimens before fixation.^32^, ^33^, ^34^, ^35^ However, specimens mounted on filter paper are more likely to be absorbed into it, thereby decreasing the sample volume required for pathological diagnosis. Therefore, in the present study, we focused on the formalin fixation process after EUS‐TA to obtain increased sample volumes and investigated the effectiveness of oil blotting paper compared to that of filter paper for formalin fixation.

## 2 METHODS

### 2.1 Study design and patients (participants)

This prospective observational study enrolled patients who underwent EUS‐TA for pancreatic tumors at the Osaka International Cancer Institute between September 2020 and February 2022. All participants were patients with suspected unresectable pancreatic cancer diagnosed via contrast‐enhanced computed tomography and who required EUS‐TA for pathological diagnosis. The clinical data of each patient were extracted from medical records. The following parameters were obtained: age, sex, tumor location, and size; EUS‐TA procedure (puncture site, needle type, and frequency [number of punctures]); complications of the EUS‐TA procedure, and histopathological diagnoses.

### 2.2 Ethical considerations

This study conformed to the Declaration of Helsinki guidelines and was approved by the Ethical Review Committee of the Osaka International Cancer Institute (Approval number: 21039–2). Written informed consent was obtained from all participants before the EUS‐TA procedure.

### 2.3 EUS‐TA procedure

All EUS‐TA procedures were performed by experienced physicians. All target lesions for EUS‐TA were pancreatic tumors that could be identified via a linear scanning video echoendoscope (GF‐UCT260; Olympus Medical Systems, Tokyo, Japan). The procedure was performed with factors such as puncture needle type, site, conditions, and frequency being appropriately considered by each attending physician. All procedures were performed in the presence of a cytopathologist who provided rapid on‐site evaluation (ROSE). The cytological samples were screened by two cytotechnologists and reviewed by an expert pathologist.

### 2.4 Filter paper and oil blotting paper

**FIGURE 1:**
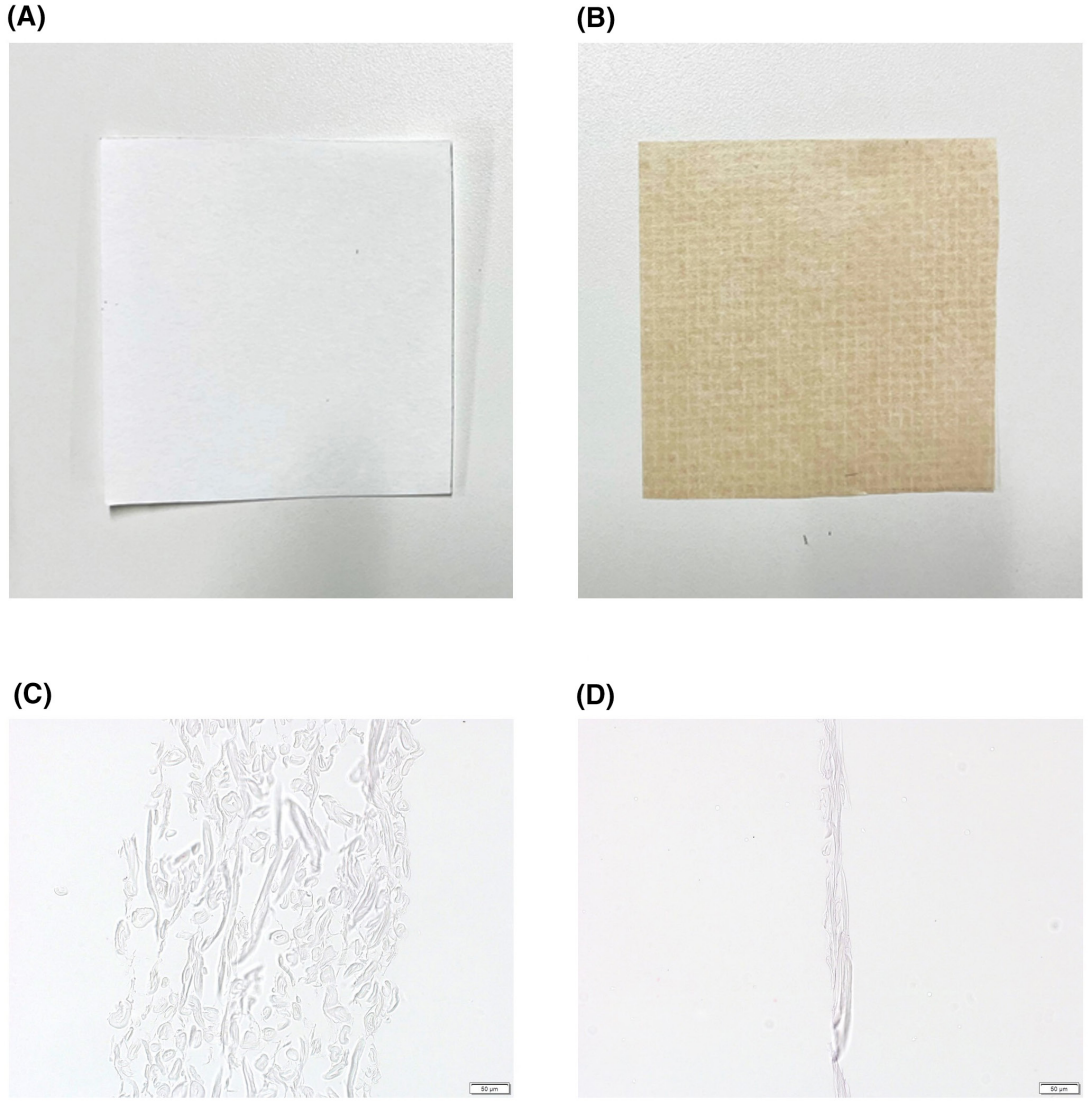
Filter paper and oil blotting paper. (A, B) The macroscopic view of filter paper (A) and oil blotting paper (B). Oil blotting paper is browner and thinner than filter paper. (C, D) The microscopic view of cross‐section after hematoxylin–eosin staining; filter paper (C) and oil blotting paper (D). Oil blotting paper is thinner, with an organized fiber‐orientation and less internal space than filter paper.

In this study, filter paper and oil blotting paper, both cut into 1.5 cm squares, were used. Macroscopically, oil blotting paper is browner and thinner than filter paper (Figure 1A,B). Microscopic images of cross sections after hematoxylin and eosin (H&E) staining revealed that the oil blotting paper was thinner and had a more organized fiber orientation and fewer internal spaces than the filter paper (Figure 1C,D).

### 2.5 Sample handling process

**FIGURE 2:**
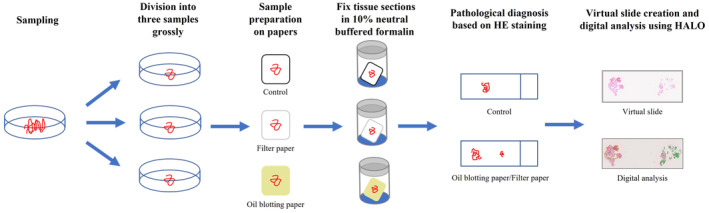
Handling process of the collected samples. EUS‐TA, endoscopic ultrasound‐guided tissue acquisition; H&E staining, hematoxylin and eosin staining.

The handling of the collected samples is illustrated in Figure 2. First, a portion of the obtained specimen was used for ROSE cytology to confirm the presence of tumor cells. Thereafter, punctures were repeated until the attending physician determined that a sufficient amount of tissue sample had been obtained. The sample from the puncture was first divided into a portion for routine histological examination. White sections were primarily used for routine histologic examination. The residual samples, primarily comprising red sections, were used for this study. The residual portion was visually divided into two groups: a filter paper group, in which specimens were spread on two pieces of filter paper and fixed in 10% neutral buffered formalin, and an oil blotting paper group, in which specimens were spread on oil blotting paper and fixed in 10% neutral buffered formalin. Because the adhesive strength of oil blotting paper is weaker than that of filter paper, specimens were sandwiched between the two firmly layered sheets of oil blotting paper and were placed statically at the bottom of the formalin bottle. Subsequently, tissue samples were prepared following the same process in both groups. Cytology and histology specimens were subjected to rapid Shorr^36^, ^37^ and H&E staining, respectively.

### 2.6 Sample analysis

**FIGURE 3:**
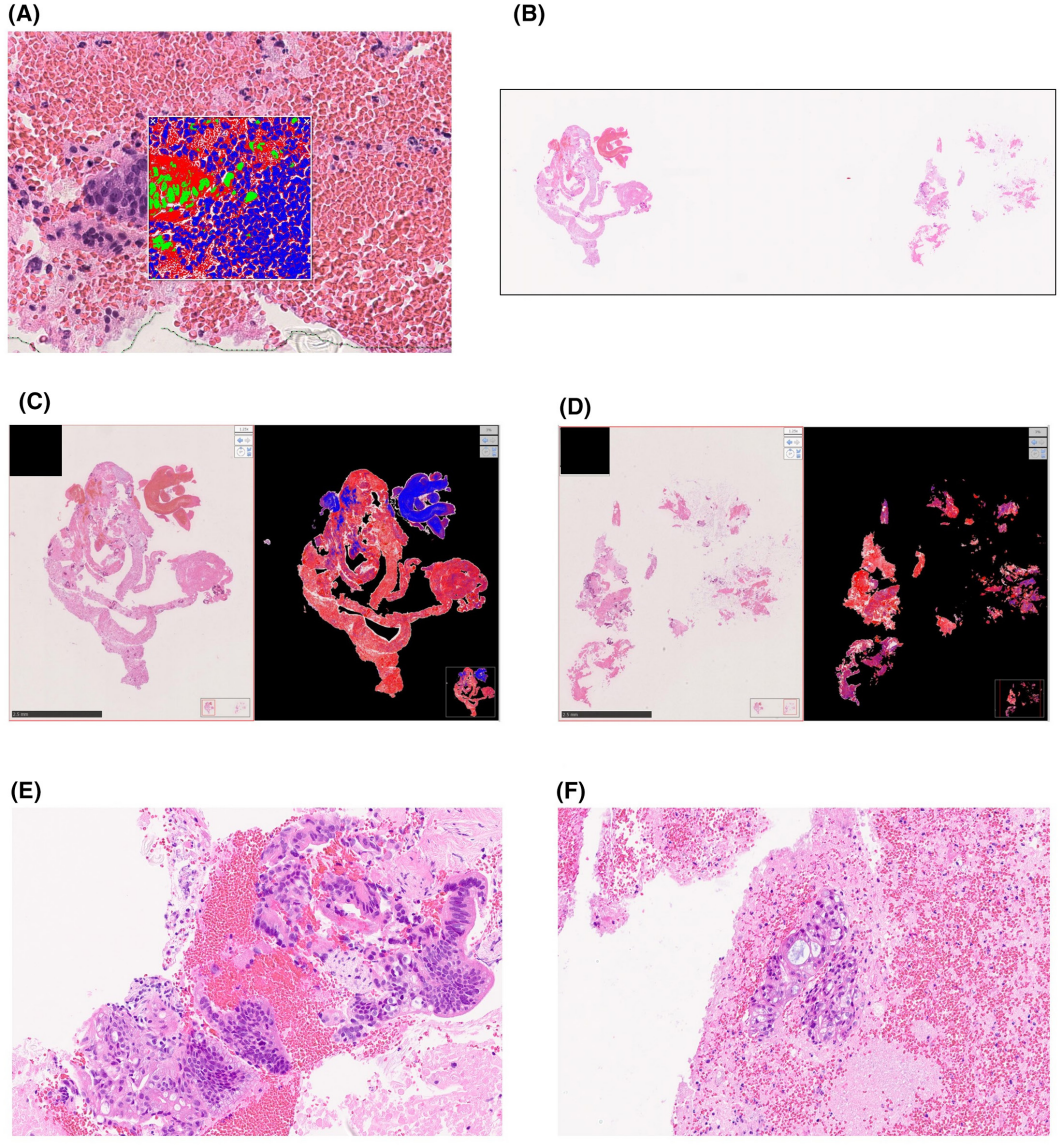
Digital analysis using HALO. (A) Definition of each the area quantified via color coding; threshold values were set using a virtual slide. Nuclei are shown in green, cytoplasm in red, and erythrocytes in blue. (B) The representative virtual slides of samples from the oil blotting paper (left) and the filter paper (right) groups. (C, D) The representative analysis of samples from the oil blotting paper (C) and filter paper (D) groups. (E, F) Representative images of large (E) and small (F) amounts of tumor cells.

Samples obtained from both groups were analyzed by the expert pathologist and his colleagues using virtual slides and visual inspection. The total tissue, nuclear, and cytoplasmic areas were calculated for each sample. Total tissue area comprised the nuclear, cytoplasmic, and erythrocyte area. Virtual slide data were created using the NanoZoomer S210 Digital slide scanner (Hamamatsu Photonics, K. K, Shizuoka, Japan) and were analyzed using HALO software (version 3.4.2986.230; Indica Labs, Corrales, NM, USA). The measurement area for each sample was defined using virtual slides; the nuclear, cytoplasm, and erythrocyte areas were specified, each threshold value was set, and each area was calculated. The nuclei are shown in green, cytoplasm in red, and erythrocytes in blue (Figure 3A). For each case, samples from the oil blotting paper group (left) and the filter paper group (right) were evaluated on the same glass slide (Figure 3B). Each area was quantified via color coding, with the thresholds set using a virtual slide of each sample (Figure 3C,D). The nuclei were shown in green, cytoplasm in red, and erythrocytes in blue (Figure 3A). For each case, samples from the oil blotting paper group (left) and the filter paper group (right) were evaluated on the same glass slide (Figure 3B). Each area was quantified by color coding with thresholds set using a virtual slide of each sample (Figure 3C,D). The sample volumes in this study differed for each case; therefore, we express the area ratios in the oil blotting paper group by assuming the area ratio of the filter paper group to be 1.0. Additionally, tumor volume evaluation and histological diagnosis were performed by the expert pathologist. Each sample was diagnosed as adenocarcinoma, suspected adenocarcinoma, or no malignancy by the expert pathologist, and tumor volume in the adenocarcinoma cases was classified as either large amount or small amount. Representative images of large and small amounts of tumoral cells are shown in Figure 3E,F.

### 2.7 Statistical analysis

Categorical variables were described as percentages and continuous variables were presented as the median and range. The diagnoses and assessments by the pathologist were compared between the filter paper and oil blotting paper methods using Fisher's exact tests. For each of the three areas (total, nuclear, and cytoplasmic), the areas in the oil blotting paper group were expressed relative to those in the filter paper group. The difference in the area ratio between the two groups was statistically analyzed with a paired *t*‐test. A *p*‐value of 0.05 was considered statistically significant. Statistical analyses were performed using the JMP software ver. 17.0 (SAS Institute, Cary, NC, USA).

## 3 RESULTS

### 3.1 Patient characteristics

Patient characteristics are summarized in Table 1. A total of 42 patients were enrolled, the median age was 71.5 years (range, 46–83), and 20 patients (47.6%) were male. The median diameter of the pancreatic tumor was 40.5 mm (range, 16–78 mm). Tumors were located in the head of the pancreas in 12 patients (28.6%). Specimens were obtained using FNA and FNB needles in 39 (92.9%) and 3 (7.1%) patients, respectively, and 22G needles were used in 39 patients (92.9%). In addition, the puncture site was through the stomach in 30 patients and through the first and second part of the duodenum in three and nine patients, respectively. The median puncture frequency was three. All patients were cytologically diagnosed with adenocarcinoma, and 36 patients (85.7%) were histologically diagnosed with adenocarcinoma. While no major complications of the EUS‐TA procedure were reported, two patients (4.8%) had minor bleeding from the puncture site; however, it resolved spontaneously without the need for blood transfusion or endoscopic hemostasis.

**TABLE 1:** Characteristics of patients with pancreatic adenocarcinoma who underwent EUS‐TA.

Number of patients, *n*	42
Median age (range), years old	71.5 (46–83)
Sex	
Male, *n* (%)	20 (47.6%)
Female, *n* (%)	22 (52.4%)
Location of target tumor	
Head, *n* (%)	12 (28.6%)
Body‐tail, *n* (%)	30 (71.4%)
Tumor diameter, median (min–max), mm	40.5 (16–78)
EUS‐FNA procedure	
Needle size	
22G, *n* (%)	39 (92.9%)
25G, *n* (%)	3 (7.1%)
Needle type	
FNA needle, *n* (%)	39 (92.9%)
FNB needle, *n* (%)	3 (7.1%)
Puncture site	
Stomach, *n* (%)	30 (71.4%)
First part of duodenum, *n* (%)	3 (7.1%)
Second part of duodenum, *n* (%)	9 (21.4%)
Number of punctures, median (min–max)	3 (1–6)
Histological diagnosis of control samples	
Cytological malignancy, *n* (%)	42 (100%)
Histological malignancy, *n* (%)	36 (85.7%)

### 3.2 Quantitative evaluation using virtual slides

Regarding the quantitative evaluation of all samples, the total, nuclear, and cytoplasmic areas were significantly larger in the oil blotting paper group than in the filter paper group (median ratio, total area: 1.39 [interquartile range 0.98–1.92], *p* = 0.003; nuclear area: 1.31 [interquartile range 0.89–2.12], *p* = 0.015; and cytoplasmic area: 1.26 [interquartile range 0.90–1.97], *p* = 0.004).

### 3.3 Visual analysis by an expert pathologist

**FIGURE 4:**
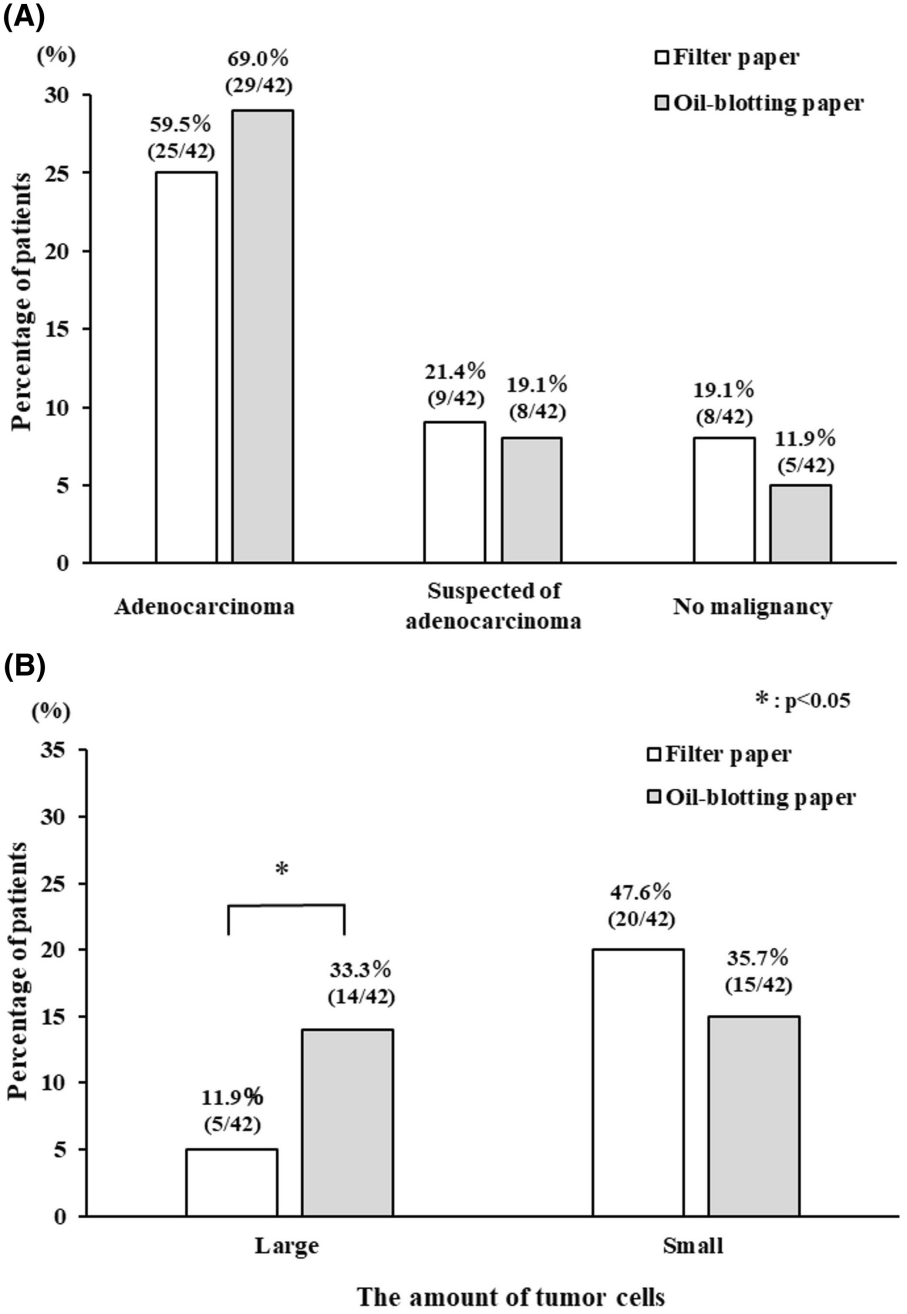
Visual analysis by an expert pathologist. Pathological diagnoses (A) and amount of tumor cells (B) were compared between the two methods using Fisher's exact tests. Differences with *p* < 0.05 were considered statistically significant.

Pathological sample diagnosis by the expert pathologist diagnosed adenocarcinoma in the samples from 29 and 25 of the patients in the oil blotting paper and filter paper groups, respectively (*p* = 0.375) (Figure 4A). No significant differences were observed between the groups in terms of suspected adenocarcinoma (9 in the filter paper group vs. 8 in the oil blotting paper group, *p* = 1.000) or absence of malignancy (eight in the filter paper group vs. five in the oil blotting paper group, *p* = 0.254). In contrast, among the adenocarcinoma cases, tumor volume was classified as large amount in five patients (11.9%) in the filter paper group and 14 patients (33.3%) in the oil blotting paper group. The frequency of cases with large amount of tumor cells was significantly higher in the oil blotting paper group (*p* = 0.035) (Figure 4B).

## 4 DISCUSSION

Pathological sampling has recently been used for the definitive diagnosis and molecular testing for initiating targeted therapies.^38^, ^39^ Although EUS‐TA is the main sampling method for the diagnosis of solid pancreatic lesions,^8^, ^40^, ^41^, ^42^ samples obtained through EUS‐TA often do not meet the suitability criteria for CGP tests.^26^ Moreover, while several studies have focused on optimal needle types and puncture methods to obtain adequate samples and on rapid and macroscopic on‐site evaluations,^15^, ^43^, ^44^, ^45^, ^46^, ^47^, ^48^ studies that focus on the formalin fixation process are scarce. In this study, we focused on the post‐sampling formalin fixation process, analyzed the diagnostic results, and compared the sample area of specimens between the filter paper and oil blotting paper methods. We found the area of the samples prepared using oil blotting paper was significantly larger than that of those prepared using filter paper.

Various methods for obtaining a larger sampling volume have been reported. Notably, FNB needles are more effective in obtaining a larger sampling volume for CGP than FNA needles.^6^, ^49^ In addition, using 19G or 22G needles at the first puncture achieves more favorable histological suitability than using a 25G needle.^25^ Although several reports have suggested that 1–4 punctures are appropriate for CGP tests,^25^, ^49^, ^50^ the present study demonstrated that the sample volume increased with the improvement of the formalin fixation process, regardless of needle type, needle thickness, or the number of punctures. This finding indicates that the oil blotting paper method may increase the available specimen volumes on the glass slide, thus improving the EUS‐TA technique.

In the present study, ROSE was performed for all patients. This technique can increase the adequacy rates and diagnostic yield of EUS‐TA by 10%–30%.^51^ Moreover, ROSE may be useful for eliminating necrotic tissue and fibrotic stroma, which cannot be removed during macroscopic on‐site evaluation, thereby improving the suitability of samples for genetic analysis.^13^ However, the routine use of ROSE with the newer‐generation FNB needles has not yet been recommended.^46^

In addition, although oil blotting papers have been used for histological examinations,^52^ to the best of our knowledge, the present study is the first to report on its efficacy for EUS‐TA. Oil blotting paper is a Japanese washi paper composed of cellulose fibers impregnated with lye or egg white to improve strength, heat resistance, surface luster, and lubricity.^53^ This paper is used as “Haku‐uchi kami” for making gold leaves.^53^ An adequately foiled paper is thin, strong, and absorbs oil and grease well; hence, this paper is also used for reapplying makeup.^54^ Filter paper has various fiber orientations and more spaces between fibers. Conversely, the fiber orientation of oil blotting paper is relatively uniform, with few spaces between fibers. The surface coating of oil blotting paper reduces the absorption of specimens into the paper. Therefore, we believe that the reason for the difference between these methods is that the sample was placed as flat and wide as possible on the oil blotting paper before formalin fixation, thus minimizing specimen loss due to absorption after formalin fixation. This approach may have resulted in an increase in the total tissue, nuclear, and cytoplasmic areas and a significant increase in the number of cases in which sufficient tissue samples were obtained among those histologically diagnosed as adenocarcinoma. Therefore, oil blotting paper may result in more tumor cells in tissue samples being available for evaluation.

In clinical practice, obtaining an adequate sample volume via EUS‐TA is important for both pathological diagnosis and molecular tests for patients with pancreatic cancer. In Japan, CGP tests using tissue samples, such as NCC Oncopanel (NOP) and FoundationOne CDx (F1CDx), have received medical insurance coverage since June 2019,^55^ and the GenMine TOP test was recently approved.^56^ The NOP analytical suitability criteria are defined as tumor cell fraction ≥20% and tissue area ≥4 mm.^57^, ^58^ Similarly, the F1 analytical suitability criteria are defined as tumor cell percentage ≥20% and tissue area ≥25 mm.^59^ Notably, the F1CDx and NOP standards were reported to be 0% and 39.2%, respectively.^26^ In clinical practice, prechecks are mandatory before CGP testing, and samples that do not pass the prechecks are not submitted. In this study, the frequency of cases with large amount of tumor cells was significantly higher in the oil blotting paper group than in the filter paper group, while no significant differences were observed in terms of pathological diagnosis. These results suggest that using oil blotting paper may improve the likelihood of CGP submission for EUS‐TA samples. Furthermore, this study is important because it focused on techniques that are yet to be standardized.

Nevertheless, this study had some limitations. First, it was a single‐center study. Second, the EUS‐TA puncture method (needle type, site, conditions, and frequency) was not unified in this study. Third, this study was performed using residual samples obtained via a single EUS‐FNA puncture. A substantial portion of each sample was used for routine histologic examination; the residual specimens were then divided into two groups (the filter paper group and the blotting paper group). Consequently, sample volume was limited in each group, and it was difficult to determine whether oil blotting paper can contribute to improving the analytical suitability criteria for CGP testing. Thus, the primary endpoint was the area of the sample rather than the actual analytical suitability criteria. Further studies are needed to compare the CGP‐testing analytical‐suitability criteria for the filter paper and oil blotting paper methods.

In conclusion, the oil blotting paper method for formalin fixation can increase the sample volume on glass slides obtained via EUS‐TA for pancreatic cancer diagnosis. This method may increase the possibility of CGP submission of EUS‐TA samples.

## AUTHOR CONTRIBUTIONS

**Takuo Yamai:** Conceptualization (lead); data curation (lead); formal analysis (lead); investigation (lead); methodology (lead); project administration (lead); resources (lead); visualization (lead); writing – original draft (lead). **Kenji Ikezawa:** Conceptualization (equal); data curation (equal); formal analysis (equal); investigation (equal); methodology (equal); project administration (equal); resources (equal); visualization (supporting); writing – original draft (supporting). **Yusuke Seiki:** Investigation (supporting); writing – review and editing (supporting). **Ko Watsuji:** Investigation (supporting); writing – review and editing (supporting). **Yasuharu Kawamoto:** Investigation (supporting); writing – review and editing (supporting). **Takeru Hirao:** Investigation (supporting); writing – review and editing (supporting). **Kazuma Daiku:** Investigation (supporting); writing – review and editing (supporting). **Shingo Maeda:** Investigation (supporting); writing – review and editing (supporting). **Makiko Urabe:** Investigation (supporting); writing – review and editing (supporting). **Yugo Kai:** Investigation (supporting); writing – review and editing (supporting). **Ryoji Takada:** Investigation (supporting); writing – review and editing (supporting). **Kaori Mukai:** Investigation (supporting); writing – review and editing (supporting). **Tasuku Nakabori:** Investigation (supporting); writing – review and editing (supporting). **Hiroyuki Uehara:** Investigation (supporting); writing – review and editing (supporting). **Sayoko Tsuzaki:** Investigation (supporting); writing – review and editing (supporting). **Ayumi Ryu:** Investigation (supporting); writing – review and editing (supporting). **Satoshi Tanada:** Investigation (supporting); writing – review and editing (supporting). **Shigenori Nagata:** Conceptualization (supporting); investigation (supporting); methodology (supporting); resources (lead); writing – review and editing (supporting). **Kazuyoshi Ohkawa:** Investigation (supporting); supervision (lead); writing – review and editing (supporting).

## FUNDING INFORMATION

None.

## CONFLICT OF INTEREST STATEMENT

Dr. Kenji Ikezawa reports honoraria for lecturing from Boston Scientific, Kaneka, Century Medical, Medicos Hirata, and J‐MIT, and consulting fees from Medicos Hirata. Dr. Ryoji Takada reports honoraria for manuscript writing from Medicos Hirata. Drs. Takuo Yamai, Yusuke Seiki, Ko Watsuji, Yasuharu Kawamoto, Takeru Hirao, Kazuma Daiku, Shingo Maeda, Makiko Urabe, Yugo Kai, Kaori Mukai, Tasuku Nakabori, Hiroyuki Uehara, Sayoko Tsuzaki, Ayumi Ryu, Satoshi Tanada, Shigenori Nagata, Kazuyoshi Ohkawa have no conflicts of interest to disclose.

## Data Availability

The data underlying the findings of our study cannot be publicly shared because of the nature of the ethical approval for the study. The data are available to researchers who meet the criteria of the Osaka International Cancer Institute Ethics Committee for access to confidential data (via email to takuo.yamai@gh.opho.jp).
